# COVID-19 in Patients with Pre-Existing Interstitial Lung Disease: Potential Value of a Steroid-Based Treatment Strategy

**DOI:** 10.3390/jcm12154940

**Published:** 2023-07-27

**Authors:** Toru Arai, Yu Kurahara, Mitsuhiro Moda, Takehiko Kobayashi, Yoshinobu Matsuda, Tomoko Kagawa, Reiko Sugawara, Kazunari Tsuyuguchi, Yoshikazu Inoue

**Affiliations:** 1 Clinical Research Center, National Hospital Organization Kinki-Chuo Chest Medical Center, Sakai City 591-8555, Osaka Prefecture, Japan; kurahara.yu.uf@mail.hosp.go.jp (Y.K.); kobayashi.takehiko.vz@mail.hosp.go.jp (T.K.); matsuda.yoshinobu.tx@mail.hosp.go.jp (Y.M.); kagawa.tomoko.qw@mail.hosp.go.jp (T.K.); tsuyuguchi.kazunari.ky@mail.hosp.go.jp (K.T.); giichiyi@me.com (Y.I.); 2 Department of Infectious Diseases, National Hospital Organization Kinki-Chuo Chest Medical Center, Sakai City 591-8555, Osaka Prefecture, Japan; 3 Department of Internal Medicine, National Hospital Organization Kinki-Chuo Chest Medical Center, Sakai City 591-8555, Osaka Prefecture, Japan; moda.mitsuhiro.qy@mail.hosp.go.jp (M.M.); fukamizu@gmail.com (R.S.)

**Keywords:** COVID-19, interstitial lung disease, treatment strategy, corticosteroids, prognosis, acute exacerbation

## Abstract

The prognosis of patients with coronavirus disease 2019 (COVID-19) and pre-existing interstitial lung disease (preILD) is poor, and no effective treatment strategy has been determined. The aim of this study was to assess the effectiveness of a steroid-based treatment strategy for patients with COVID-19 and preILD. We retrospectively reviewed the medical records of 610 consecutive patients with COVID-19 treated at our institution between 1 March 2020 and 30 October 2021 and identified 7 patients with preILD, all of whom were treated with corticosteroids and remdesivir. All the patients were men with a median age of 63 years. Three of four patients with severe disease required invasive positive-pressure ventilation (*n* = 2) or nasal high-flow therapy (*n* = 1). All three patients could be weaned from respiratory support; however, one died in hospital. The remaining patient with severe COVID-19 had a do-not-resuscitate order in place and died while hospitalized. All three patients with moderate COVID-19 were discharged. The 30-day mortality was 0%, and the mortality rate during the entire observation period was 28.5%. The prognosis of our patients with COVID-19 and preILD has been better than in previous reports. Our management strategy using corticosteroids may have improved these patients’ prognosis.

## 1. Introduction

Coronavirus disease 2019 (COVID-19) is caused by severe acute respiratory syndrome coronavirus 2 (SARS-CoV-2) and mainly affects the lung parenchyma [1,2]. Patients with interstitial lung disease (ILD) who develop COVID-19 are reported to have poor outcomes [3,4,5,6]. According to the RECOVERY trial, dexamethasone improves the prognosis of patients with COVID-19 who require supplemental oxygen [7]. There are some data suggesting that SARS-CoV-2 infection may trigger acute exacerbation of ILD (AE-ILD) in patients with pre-existing ILD (preILD) [8,9,10,11]. Hence, the treatment strategy for AE-ILD, namely, the use of high-dose corticosteroids [12] with a tapering of doses over several months, may be effective in patients who are hospitalized with COVID-19 and preILD and who fulfilled the diagnostic criteria of AE-ILD [12,13]. The purpose of this study was to assess the effectiveness of this treatment strategy in patients with COVID-19 and preILD at our institution. 

## 2. Materials and Methods

This study was approved by the National Hospital Organization Kinki-Chuo Chest Medical Center’s Institutional Review Board (approval number Rin2021-086, date of approval: 3 February 2022) and was performed in accordance with the Declaration of Helsinki. The requirement to obtain written informed consent was waived due to this study’s retrospective design.

We retrospectively reviewed the medical records of 610 consecutive patients with COVID-19 who were treated at our institution between 1 March 2020 and 30 October 2021; we identified 7 patients from this sample with preILD. Our facility is designated for the management of patients with COVID-19 with mild-to-moderate symptoms. Patients who deteriorate to the point of requiring mechanical ventilation are usually transferred to a hospital designated for the treatment of patients with severe COVID-19 symptoms. Recovered patients who are completely weaned from mechanical ventilation usually come back to our hospital. 

### 2.1. Diagnosis of PreILD

In all seven cases, ILD had been diagnosed before the onset of COVID-19. The diagnosis of ILD was based on the diagnostic criteria for the different types of ILD, such as idiopathic interstitial pneumonias (IIPs) [14] and idiopathic pulmonary fibrosis (IPF) [15]. Patients with IIPs except for IPF diagnosed without surgical lung biopsy (SLB) were classified as unclassifiable ILD (UN-ILD). IPF, in which pathological usual interstitial pneumonia (UIP) was confirmed by SLB specimens, was described as IPF/UIP. ILD complicated by collagen vascular disease was diagnosed according to the specific diagnostic criteria for each type of collagen vascular disorder [16,17,18]. Hypersensitivity pneumonitis was not considered a preILD in this study. 

### 2.2. Severity of COVID-19

The severity of COVID-19 was defined according to the guidelines for its management in Japan [19] and classified as mild (not requiring supplemental oxygen), moderate (with pneumonia and/or requiring supplemental oxygen), or severe (requiring mechanical ventilation and/or extracorporeal membrane oxygenation or admission to the intensive care unit).

### 2.3. Treatment Strategy for COVID-19 with PreILD

All patients with COVID-19 and preILD who fulfilled the diagnostic criteria for AE-ILD [12,13] were treated with corticosteroids and remdesivir as an antiviral agent [20]. High-dose corticosteroids, usually methylprednisolone at a dose of 500–1000 mg/day for three successive days, followed by 0.5–1.0 mg/kg of prednisolone, were administered according to the severity or extent of ground-glass opacities. Dexamethasone was administered before prednisolone in some cases. The prednisolone dose was usually reduced to half of the starting dose within 2 or 3 months and tapered off according to the clinical course of the illness. In our clinical practice, the treatment strategy for COVID-19 with preILD is similar to that for AE-ILD [12], although immunosuppressants, including cyclosporine A, azathioprine, and tacrolimus, were not usually administered for COVID-19 with preILD. Some patients with COVID-19 were treated with baricitinib (oral administration), a selective inhibitor of Janus kinase 1 and 2, as an immunomodulatory drug coupled with remdesivir [21]; however, barcicinib was not used for the treatment of AE-ILD. 

### 2.4. Diagnosis of SARS-CoV-2

Diagnosis of COVID-19 was based on a polymerase chain reaction test or an antigen test for SARS-CoV-2 from saliva, sputum, or nasopharyngeal swabs [2]. Strains of SARS-CoV-2 in each patient were not confirmed; however, we could suspect the strains (wild type, alpha strain, or delta strain) according to their epidemiological frequency at the disease onset [22,23,24,25]. 

### 2.5. Data Collection

Using the patients’ electronic medical records, we collected data on age; sex; body weight; height; body mass index; disease severity; smoking history; comorbidities; and laboratory data (including lactate dehydrogenase, ferritin, and Krebs von den Lungen-6 levels) at admission. Pulmonary function test results, including the percent predicted forced vital capacity (%FVC) and percent predicted diffusing capacity of carbon monoxide (%DLco), high-resolution computed tomography (HRCT) findings, and data on the introduction of long-term oxygen therapy before and after the onset of COVID-19, were also collected. HRCT patterns of preILDs in each patient before the onset of COVID-19 were evaluated according to the IPF guidelines [15].

We also collected treatment details, including the management of ventilation, and outcomes data. Serum Krebs von den Lungen-6 levels were measured using commercial enzyme-linked immunosorbent assay kits (Eizai, Tokyo, Japan) with a cut-off level of 500 U/mL [26]. HRCT patterns of preILDs were evaluated according to the international guideline of IPF [15]. 

### 2.6. Description of the Clinical Course

The time course of the respiratory support, steroid treatment, and outcome, including the hospital stay and survival period, were recorded. The clinical courses of severe cases and relapsed cases were explained coupled with HRCT findings. However, the changes in HRCT findings in severe and deceased cases could not be evaluated. 

### 2.7. Comparison of the Clinical Course between Our Study and Previous Studies

We compared the outcome between patients with COVID-19 and preILD in our study and that in other reports [3,4,5,6]. The subjects of two previous studies [3,4] were in-hospital patients with COVID-19 and preILD and those of the other two previous studies were all patients with COVID-19 and preILD [5,6]. In the latter studies, the mortality rate, 30-day mortality rate, and frequency of corticosteroid-treated in-patients were calculated, and it was hypothesized that all such patients were hospitalized. Statistical analysis was not performed because our study included a limited number of patients. 

## 3. Results

### 3.1. Patient Demographics

**Figure 1:**
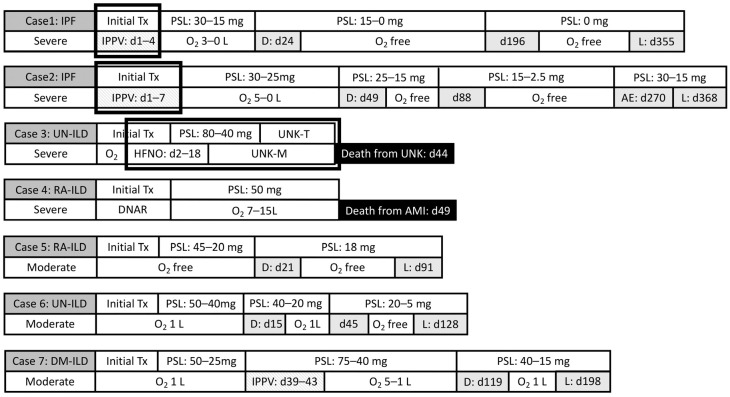
Clinical course of seven patients with COVID-19 and pre-existing interstitial lung diseases: respiratory support and steroid therapy. Abbreviations: AE, acute exacerbation; AMI, acute myocardial infarction; D, discharge; d, day; DM, dermatomyositis; DNAR, do not attempt resuscitation; ILD, interstitial lung disease; IPF, idiopathic pulmonary fibrosis; IPPV, invasive positive-pressure ventilation; L, last follow-up; HFNO, high-flow nasal oxygen; PSL, prednisolone; RA, rheumatoid arthritis; UC, unclassifiable; UNK, unknown cause; UNK-S, unknown respiratory support; UNK-T, unknown steroid therapy. Initial Tx means initial steroid therapy including methylprednisolone pulse therapy and post-pulse steroid therapy, as shown in Table 2. Periods during which Cases 1, 2, and 3 were treated for intensive respiratory support in the other hospitals are shown by bold squares.

Seven patients with COVID-19 had preILD (Table 1, Figure 1). All the patients were men with a median age of 63 years (range, 49–77). Three patients were non-smokers, three were ex-smokers, and one was a current smoker. Cardiovascular diseases and emphysema were co-occurring in three and four patients, respectively; however, active malignant diseases or diabetes mellitus were not diagnosed in any patients. The preILD was IPF in two cases; unclassifiable IIP in two; and ILD associated with collagen vascular disease in three (rheumatoid arthritis, *n* = 2; dermatomyositis, *n* = 1). 

PreILD was treated with corticosteroids and/or immunosuppressive agents in three patients and an antifibrotic drug, nintedanib, in one patient before the onset of COVID-19. Long-term oxygen therapy had been introduced in the patient with preILD associated with dermatomyositis before the onset of COVID-19 (Table 1). Pulmonary function tests were performed in all cases except for one of the two patients with rheumatoid arthritis, and the median predicted percent forced vital capacity (%FVC) before the onset of COVID-19 was 91.1%.

At the onset of COVID-19, C-reactive protein and lactate dehydrogenase levels were elevated in all patients. Serum ferritin levels were elevated in five out of seven patients (Table 1). 

### 3.2. Severity of COVID-19 and Treatment

COVID-19 symptoms were severe in four patients and moderate in three. All patients fulfilled the diagnostic criteria for AE-ILD [12,13] and received remdesivir as an antiviral agent and corticosteroids as immunosuppressive therapy. Five of the seven patients received high-dose intravenous corticosteroid therapy (methylprednisolone ≥ 500 mg). Five patients received dexamethasone and the other two patients received a tapered dose of methylprednisolone (125 mg daily). After this therapy, all patients received oral prednisolone (more than 30 mg daily). Baricitinib was administered to one patient with moderate symptoms of COVID-19 (Table 2).

### 3.3. Outcomes

**Figure 2:**
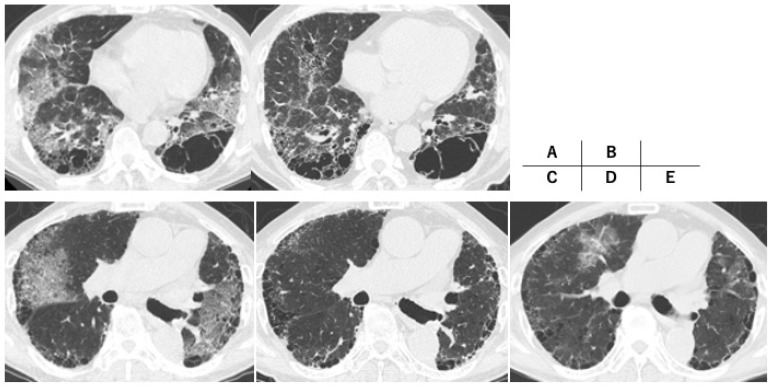
High-resolution computed tomography findings of severe cases. Case 1 (**A**,**B**): idiopathic pulmonary fibrosis (IPF), a 67-year-old man ((**A**): onset of COVID-19, (**B**): one year after onset of COVID-19); Case 2 (**C**–**E**): IPF, a 73-year-old man ((**C**): onset of COVID-19, (**D**): five months after onset of COVID-19, (**E**): acute exacerbation nine months after onset of COVID-19).

One of the four patients with severe COVID-19 (Case 4) had a do-not-resuscitate order in place and was managed by standard oxygen inhalation (Figure 1). Two of the remaining three patients with severe COVID-19 required invasive positive-pressure ventilation, which was able to be withdrawn in both cases (Figure 2). These two patients were discharged after the tapering of their prednisolone dose and discontinuation of supplemental oxygen; the third patient improved after 16 days of nasal high-flow oxygen therapy but died suddenly of an unknown cause on day 44 of in-patient treatment. The patient with the do-not-resuscitate order died of acute myocardial infarction on day 49 (Figure 1).

**Figure 3:**
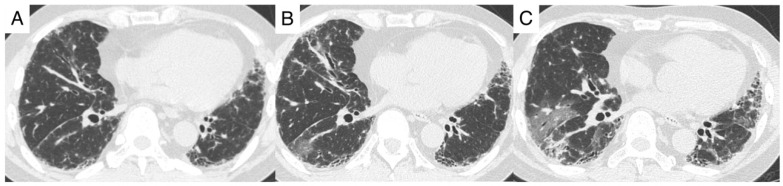
High-resolution computed tomography findings of Case 7. Dermatomyositis interstitial lung diseases, a 53-year-old man ((**A**): three months before onset of COVID-19, (**B**): onset of COVID-19, (**C**): one month after onset of COVID-19).

All three patients with moderate COVID-19 improved rapidly after the initiation of corticosteroid therapy (Table 2, Figure 1). At discharge, one of these patients required supplemental oxygen at a rate of 1 L/min. The patient with ILD as a complication of dermatomyositis developed ground-glass opacities involving both lungs and a relapse of hypoxemia approximately one month after the onset of COVID-19 (Figure 1 and Figure 3). This patient’s condition improved after further treatment with corticosteroids and invasive positive-pressure ventilation (Figure 1). 

All seven patients survived for at least 30 days, and both the 60-day mortality rate and the mortality rate during the entire observation period were 28.5% (Table 2). FVC was evaluated before and after COVID-19 in four patients, all of whom had a decline of >5% (Table 3). Reticular shadows increased after COVID-19 (Figure 2 and Figure 3), although it was unclear whether this radiological change reflected the natural course of preILD or was a sequela of COVID-19. None of the seven patients had an infection or diabetes mellitus after the induction of steroid therapy. 

### 3.4. Comparison with Other Studies

We summarized the details of the four studies [3,4,5,6] and our study and compared the seven patients with COVID-19 and preILDs with those of other studies (Table 4). The in-hospital mortality rate of our study (28.5%) was lower than that of the studies of Drake et al. [4] (49.1%) and Esposito et al. [5] (44.1%).

## 4. Discussion

Thus far, we have treated seven patients with a diagnosis of COVID-19 and preILD. All patients showed transient improvement, and five were discharged from the hospital alive. Previous studies have found that patients with COVID-19 and preILD have a poor prognosis and a high mortality rate [3,4,5,6,10]. However, the outcomes in our cohort have not been as bleak as previously reported. 

Several studies have identified preILD to be a risk factor for developing a severe outcome of COVID-19 [27]. Drake et al. reported that 49.1% of their patients with COVID-19 and preILD died while hospitalized [4]. Another study reported a 30-day mortality rate of 35% for in-patients with COVID-19 and preILD [6]. Naqvi et al. reported 30-day and 60-day mortality rates of 27.93% and 30.63%, respectively, in in-patients with COVID-19 and IPF [3]. Esposito et al. found that 34 of 46 patients with COVID-19 and ILD required admission to the hospital and that 15 (44.1%) of these hospitalized patients died [5]. In contrast with those reports, our 30-day and 60-day mortality rates in patients with COVID-19 and preILD were 0% and 28.5%, respectively. The mortality rate during the entire observation period was also 28.5%. 

Although our experience is based on a limited number of patients, these patients’ prognoses seem better than those reported elsewhere [4,5,6]. This difference in outcome might reflect the fact that our patients with COVID-19 and preILD received frequent doses of corticosteroids in addition to remdesivir, whereas fewer such patients in the previous reports received corticosteroids. In addition, the previous reports mainly included patients before the approval of remdesivir by the U.S. Food and Drugs Administration in October, 2020 [28]. Gallay et al. [6] and Esposito et al. [5] reported that approximately 10% of their patients with COVID-19 and preILD were treated with corticosteroids for COVID-19. Drake et al. [4] reported that 45 of 161 in-hospital patients with COVID-19 and preILD (28.0%) were treated with corticosteroids and their mortality rate (48.8%) was clearly worse than our study (28.5%). Although the dose and duration of corticosteroid treatment was not clarified in their study, our steroid-based treatment strategy might be better than the steroid treatment of Drake et al. [4]. Kondoh et al. reported a 30-day mortality of 50% in 12 patients with COVID-19-associated AE-ILD [10]; however, the participating institutions in their study did not provide information on the severity of COVID-19, and the clinical parameters and treatment—including possible corticosteroid use—were not described in detail. 

New ground-glass opacities and an elevated oxygen requirement in patients with COVID-19 and preILD suggest a diagnosis of AE-ILD [13]. Acute deterioration of respiratory status in association with influenza in patients with ILD is diagnosed as AE [29] in view of the rarity of influenza-associated pneumonia [30]. COVID-19 also has a high mortality rate in patients without ILD [1,2]; thus, COVID-19 can be treated simply as a complication in patients with preILD. Therefore, whether acute deterioration caused by an apparent infection can be accurately called “triggered AE-ILD” is controversial [8]. However, AE-ILD and COVID-19 have some pathophysiological similarities. Accelerated alveolar epithelial apoptosis is thought to be the main pathophysiological mechanism in AE-ILD [15]. SARS-CoV-2 infection occurs via angiotensin-converting enzyme receptors, and alveolar apoptosis is a feature of COVID-19 [31]. The cytokine dynamics associated with prognosis are similar between AE-IIP [32] and COVID-19 with preILD [33,34]. Therefore, it seems likely that a management strategy for COVID-19 with preILD that is similar to that for AE-ILD could improve survival, regardless of whether these two diagnoses are the same entity.

In the RECOVERY trial [7], the 28-day mortality rate was lower in patients who received dexamethasone for up to 10 days in addition to usual care than in those who received usual care alone. Patients with COVID-19 are usually treated with dexamethasone and an antiviral agent, typically remdesivir. In our hospital, we usually treat AE-ILD with intravenous high-dose methylprednisolone—so-called “pulse therapy”—followed by a maintenance dose of prednisolone that is gradually tapered [12]. In patients with COVID-19 and preILD, we have used dexamethasone after pulse therapy and then switched to prednisolone. Immunosuppressants, including cyclosporine A, azathioprine, and tacrolimus [12,35,36], which are often used for AE-ILD [12], are not used in patients with COVID-19 and preILD at our institution. However, these agents may be useful in view of a report by Gálvez-Romero et al. indicating that cyclosporine A plus low-dose steroids improves the survival rate in patients with COVID-19 [37]. 

There may be some disagreements regarding the treatment strategy for COVID-19, in particular the value of long-term steroid therapy. Fonseca et al. published a report on a patient with rheumatoid-arthritis-associated ILD and COVID-19, which relapsed after approximately one week of steroid therapy and needed non-invasive positive-pressure ventilation [38]. Case 7 in our series also experienced a relapse requiring invasive positive-pressure ventilation one month after onset of COVID-19, purportedly because of a rapid reduction in the patient’s steroid dose. Patients without ILD who develop COVID-19 show persistent inflammatory abnormalities after the acute period, and post-COVID interstitial fibrosis in the chronic phase is an important problem [39]. Myall et al. suggested that prednisolone should be used at a maximum initial dose of 0.5 mg/kg for persistent inflammatory ILD that lasts for more than a month after COVID-19 [40]. Their report recommended a tapering of the prednisolone dose over approximately three weeks. In our Case 1, we tapered the prednisolone dose over approximately six months. However, the appropriate treatment duration for corticosteroids in patients with COVID-19 and preILD remains to be determined. 

The progression of pulmonary fibrosis after COVID-19 in patients with preILD is another problem. We could not compare the exact FVC before and after COVID-19 because FVC was not evaluated immediately before the onset of the illness; however, there were definite decreases in FVC (Table 2) and in the radiographically confirmed progression of fibrotic lesions in our cases. The early administration of an antifibrotic agent might be able to inhibit late-phase COVID-related fibrosis after steroid therapy [41]. 

We managed our patients with COVID-19 and preILD using our standard treatment strategy for AE-ILD; however, the prognosis of patients with COVID-19 and preILD may be better than that of patients with AE-ILD or AE-IIP. We previously reported a 30-day mortality rate of 40% (34/85) in patients with AE-IIP [12], and Suzuki et al. found a 30-day mortality rate of 23.8% (46/193) in patients with AE-ILD [42]. In contrast, the 30-day mortality rate in patients with COVID-19 and preILD in our study was 0%. This finding might reflect differences in the histological features of AE-ILD and COVID-19. In both diseases, the main finding in the lungs at autopsy is diffuse alveolar damage [43,44]. Churg et al. also reported that surgical lung biopsy specimens showed diffuse alveolar damage (*n* = 4), organizing pneumonia (OP) (*n* = 5), and fibrosis (*n* = 3) [45]. Lung biopsy specimens cannot be obtained easily from patients with COVID-19 because of the risk of infection to others; however, Doglioni et al. recently reported that none of their cryobiopsy specimens, mostly obtained within two weeks after symptom onset, showed a typical pattern of diffuse alveolar damage [46]. Pogatchnik et al. reported that patients with COVID-19 whose transbronchial lung biopsy specimens showed an organizing pneumonia (OP) pattern had a good prognosis [47]. Kory et al. suggested that the prevalence of OP in early disease might be higher than previously reported [48]. Hence, the difference in frequency of the OP pattern might explain the better short-term outcome in our patients than in their counterparts with AE-ILD despite the use of the same treatment strategy. 

This study has several limitations. First, it had a single-center, retrospective observational design and included a small number of cases. We have insufficiently compared the survival of our COVID-19 patients with preILD with that of the patients in the previous reports. In addition, our treatment strategy was quite different from the current standard strategy of dexamethasone therapy for COVID-19. Therefore, a large-scale multicenter study comparing dexamethasone treatment and our treatment strategy is needed to reach definitive conclusions. Second, the higher %FVC in six of our patients with preILD before the onset of COVID-19 might have contributed to the survival rate of patients with COVID-19 and preILD being better than in previous reports. Drake et al. [4] reported that a %FVC > 80% suggests a greater likelihood of survival in patients with preILD. Third, preILD in this study comprised only two cases of IPF, which might have led to these patients’ better prognosis of COVID-19 with preILD. However, Suzuki et al. reported similarly poor prognoses in patents with AE of IPF or other fibrosing types of ILD [40]. Fourth, this study did not include patients with COVID-19 and other pre-existing interstitial lung abnormalities [49]. Fifth, this study did not include cases of COVID-19 caused by the Omicron variant [50] and preILD, and the treatment strategy for such cases needs to be evaluated in another study because Omicron infection has been associated with lower mortality and hospitalization [51]. 

## 5. Conclusions

Out of 610 consecutive patients with COVID-19 diagnosed at our institution, 7 also had preILD. All seven of these patients were treated with long-term corticosteroids using the management strategy for AE-ILD in addition to remdesivir. The prognosis of these patients might have been better than that described in previous reports, although large-scale prospective studies are needed to draw definite conclusions about the efficacy of our management strategy. 

## Figures and Tables

**Table 1:** Demographics of patients with COVID-19 and preILD.

	Case 1	Case 2	Case 3	Case 4	Case 5	Case 6	Case 7
Severity of COVID-19	Severe	Severe	Severe	Severe	Moderate	Moderate	Moderate
Suspected strain of SARS-CoV-2 *	Wild type	Wild type	Alpha	Alpha	Wild type	Delta	Alpha
Sex	Male	Male	Male	Male	Male	Male	Male
Age	67	73	63	77	61	49	53
Weight, Kg	67.7	75.6	100.3	50	67.0	80.8	69.4
Height, m	1.694	1.777	1.747	1.540	1.605	1.790	1.730
BMI	23.6	23.9	32.8	21.5	26.0	25.2	23.2
Smoking	ES	ES	NS	NS	CS	ES	ES
PreILDs	Clinical IPF	IPF/UIP	UN-ILD	RA-ILD	RA-ILD	UN-ILD	DM-ILD
CT pattern of preILDs	UIP	UIP	Indeterminate for UIP	NA	Alternative	Alternative	Alternative
%FVC **	88.6%	109.5%	93.6%	NA	110.1%	86.1%	57.0%
%DLco **	81.0%	92.5%	85.6%	NA	122.5%	43.6%	39.0%
Cardiovascular diseases	Atrial fibrillation	No	No	HT, OMI	No	No	HT
Emphysema	Yes	Yes	No	Yes	No	Yes	No
Other lung diseases	No	Post LC operation	No	No	No	No	No
Hyperlipidemia	No	No	No	No	Yes	Yes	No
Other comorbidities ^¶^	n.p.	Chronic sinusitis	Spinal canal stenosis	n.p.	Fatty liver	n.p.	n.p.
Tx for ILDs before COVID-19	No	No	No	TCZ, SASP	MTX, PSL 7.5 mg/day	No	PSL, CyA, Nintedanib
LTOT before COVID	No	No	No	No	No	No	Yes
**COVID-19 onset**							
CRP, mg/dL	31.97	5.72	8.16	3.03	9.72	3.49	4.13
LDH, IU/mL	507	458	709	623	402	254	371
KL-6, U/mL	1254	1334	2069	2306	563	1285	905
Ferritin, ng/mL	NA	743.5	698.1	1066.9	2057.2	376.9	88.6

Abbreviations: BMI, body mass index; COVID-19, coronavirus disease 2019; CRP, C-reactive protein; CS, current smoker; CyA, cyclosporine A; DM, dermatomyositis; ES, ex-smoker; FVC, forced vital capacity; HT, hypertension; ILD, interstitial lung disease; IPF, idiopathic pulmonary fibrosis; KL-6, Krebs von den Lungen-6; LC, lung cancer; LDH, lactate dehydrogenase; LTOT, long-term oxygen therapy; MTX, methotrexate; NA, not available; NS, non-smoker; OMI, old myocardial infarction; preILD, pre-existing ILD; PSL, prednisolone; RA, rheumatoid arthritis; SASP, salazosulfapyridine; TCZ, tocilizumab; Tx, therapy; UIP, usual interstitial pneumonia; UN, unclassifiable. *: Strains of SARS-CoV-2 in each patient were not confirmed by polymerase chain reaction; however, we could suspect the strains according to their epidemiological frequency at disease onset. **: Pulmonary function tests were performed within one year from the onset of COVID-19. ^¶^: None of the seven patients had comorbid pulmonary hypertension, gastroesophageal reflux disease, active malignant diseases, or diabetes mellitus.

**Table 2:** Treatment of patients with COVID-19 and preILD.

	Case 1	Case 2	Case 3	Case 4	Case 5	Case 6	Case 7
Severity of COVID	Severe	Severe	Severe	Severe	Moderate	Moderate	Moderate
Vaccination	No	No	No	No	No	No	No
Antiviral drugs	RMD	RMD	RMD	RMD	RMD	RMD	RMD
mPSL pulse Tx *	Yes, 1000 mg/day	Yes, 1000 mg/day	Yes, 1000 mg/day	Yes, 500 mg/day	No	Yes, 500 mg/day	No
Post-pulse Tx *	DEX 6 mg/day	DEX 6 mg/day	DEX 6 mg/day	DEX 3.3 mg/day	DEX 8 mg/day	mPSL 125 mg for 3 days, 80 mg for 4 days	mPSL 125 mg for 3 days, 60 mg for 3 days,
Maintenance steroid Tx	PSL 30 mg/day	PSL 30 mg/day	PSL 80 mg/day	PSL 50 mg/day	PSL 45 mg/day	PSL 50 mg/day	PSL 50 mg/day
Other anti-inflammatory drugs	No	No	No	No	No	Baricitinib	No
ICU entry	Yes	Yes	Yes	No	No	No	No
Maximum respiratory support	IPPV	IPPV	HFNO	Supplemental oxygen, DNAR	Supplemental oxygen	Supplemental oxygen	Supplemental oxygen
Discharge alive	Yes	Yes	No	No	Yes	Yes	Yes
Hospital stay, days	24	49	44	49	21	15	119
Duration of steroid Tx **, days	196	368	44	49	91	128	198
Final observation	Alive	Alive	Dead	Dead	Alive	Alive	Alive
Observation, days	355	368	44	49	91	128	198

Abbreviations: COVID-19, coronavirus disease 2019; DNAR, do not attempt resuscitation; ICU, intensive care unit; ILD, interstitial lung disease; IPF, idiopathic pulmonary fibrosis; IPPV, invasive positive-pressure ventilation; LTOT, long-term oxygen therapy; mPSL, methylprednisolone; MTX, methotrexate; HFNO, high-flow nasal oxygen; NS, non-smoker; preILDs, pre-existing ILDs; PSL, prednisolone; RA, rheumatoid arthritis; RMD, remdesivir; SASP, salazosulfapyridine; TCZ, tocilizumab; Tx, therapy; UIP, usual interstitial pneumonia. * Initial Tx in Figure 1 means initial steroid therapy including methylprednisolone pulse therapy and post-pulse steroid therapy. **: Case 1 improved and steroid therapy could be stopped. Case 3 and Case 4 died and steroid administration ended.

**Table 3:** Pulmonary function test (FVC) before and after onset of COVID-19.

	Before COVID-19 *	After COVID-19	Interval from COVID-19 Onset to PFT
Case 1	3.02 (88.6%)	2.54 (74.9%)	12 months
Case 2	3.81 (109.5%)	2.99 (87.7%)	5 months
Case 6	3.41 (86.1%)	3.08 (78.4%)	6 months
Case 7	2.13 (57.0%)	1.62 (43.5%)	3 months

Abbreviations: FVC, forced vital capacity; COVID-19, coronavirus disease 2019; PFT, pulmonary function test. FVC was shown as Litter (%predicted FVC); *: Pulmonary function tests were performed within one year from the onset of COVID-19.

**Table 4:** Outcome of patients with COVID-19 and preILD in our study and other studies.

Studies	Our Study	Naqvi [3]	Drake [4]	Esposito [5]	Gallay [6]
No. of patients	7	111	161	46	123
Type of ILDs	ILDs *	IPF	ILDs *	ILDs *	ILDs *
In-hospital patients, No.	7	111	161	34	103
Respiratory support, No. (%)	0 (0)	NA	156 (96.9) **	NA	NA
ICU level of care, No. (%)	3 (42.8) **	NA	20 (12.4) **	16 (47.0) **	26 (25.24) **
Mortality, No. (%)	2 (28.5) **	NA	79 (49.1) **	15 (44.1) ** ^¶^	NA
30-day mortality, No. (%)	0 (0) **	31 (27.93) **	NA	NA	31 (30.1) ^§^
60-day mortality, No. (%)	2 (28.5) **	34 (30.63) **	NA	NA	NA
Corticosteroids for COVID-19, No. (%)	7 (100) **	NA	45 (28.0) **	4 (11.8) ^#^	14 (13.6) ^†^
Mortality of corticosteroid-treated patients, No. (%)	2 (28.5)	NA	22 (48.8)	1 (25.0)	NA

Abbreviations: COVID-19, coronavirus disease 2019; ICU, intensive care unit; ILD, interstitial lung disease; IPF, idiopathic pulmonary fibrosis; preILDs, pre-existing ILDs; No., number. *: IPF and other ILDs. **: Out of all in-hospital patients with COVID-19 and preILD. ^¶^: Originally, it was described that 15 out of 46 patients with COVID-19 and preILD (32.61%) died. Mortality of in-patients was calculated and it was hypothesized that all dead patients were hospitalized. ^§^: Originally, it was described that the 30-day mortality rate was 31 out of 123 patients with COVID-19 and preILD (25.0%). The 30-day mortality of in-patients (*n* = 103) was calculated and it was hypothesized that all such patients were hospitalized. ^#^: Originally it was described that 4 out of 46 patients with COVID-19 and preILD (8.6%) were treated with corticosteroids. The frequency of corticosteroid treatment in in-patients was calculated and it was hypothesized that all such patients were hospitalized. ^†^: Originally it was described that 14 out of 123 patients with COVID-19 and preILD (11.4%) were treated with corticosteroids. The frequency of corticosteroid administration in in-patients was calculated and it was hypothesized that all such patients were hospitalized.

## Data Availability

The data supporting the findings of this study are available from the corresponding authors upon reasonable request, if ethical and legal concerns are not present.
